# Effects of Resveratrol on p66Shc phosphorylation in cultured prostate cells

**Published:** 2016-01-31

**Authors:** A. Conte, C. Procaccini, P. Iannelli, A. Kisslinger, F. De Amicis, G.M. Pierantoni, F.P. Mancini, G. Matarese, D. Tramontano

**Affiliations:** 1 Department of Molecular Medicine and Medical Biotechnologies, University of Naples “Federico II”, Naples, Italy;; 2 Institute of Endocrinology and Experimental Oncology, CNR, Naples, Italy.; 3 Department of Pharmacy, Health and Nutritional Sciences, University of Calabria, Arcavacata di Rende (CS), Italy; Centro Sanitario, University of Calabria, Arcavacata di Rende (CS), Italy;; 4 Department of Sciences and Technologies, University of Sannio, Benevento, Italy;; 5 Fondazione GENS Onlus, Naples Italy.

**Keywords:** Resveratrol, Shc, prostate, age-related diseases

## Abstract

There is increasing evidence that diet plays a crucial role in age-related diseases and cancer. Oxidative stress is a conceivable link between diet and diseases, thus food antioxidants, counteracting the damage caused by oxidation, are potential tools for fight age-related diseases and cancer.

Resveratrol (RSV), a polyphenolic antioxidant from grapes, has gained enormous attention particularly because of its ability to induce growth arrest and apoptosis in cancer cells, and it has been proposed as both chemopreventive and therapeutic agent for cancer and other diseases. Even though the effects of RSV have been studied in prostate cancer cells and animal models, little is known about its effects on normal cells and tissues. To address this issue, we have investigated the effects of RSV on EPN cells, a human non-transformed prostate cell line, focusing on the relationship between RSV and p66Shc, a redox enzyme whose activities strikingly intersect those of RSV. p66Shc activity is regulated by phosphorylation of serine 36 (Ser36) and has been related to mitochondrial oxidative stress, apoptosis induction, regulation of cell proliferation and migration. Here we show that RSV inhibits adhesion, proliferation and migration of EPN cells, and that these effects are associated to induction of dose- and time-dependent p66Shc-Ser36 phosphorylation and ERK1/2 de-phosphorylation. Moreover, we found that RSV is able to activate also p52Shc, another member of the Shc protein family. These data show that RSV affects non-transformed prostate epithelial cells and suggest that Shc proteins may be key contributors of RSV effects on prostate cells.

## I. INTRODUCTION

Health or disease is shaped for all individuals by interactions between genes and environment and, among environmental factors, nutrition plays a key role in preventing or causing diseases. The most important message of modern research in nutrition is that a diet rich in fruit, vegetables, legumes, and whole grains, and which includes fish, nuts and low-fat dairy products, protects against almost all non-communicable diseases and aging [1]. The richness in food antioxidants, which fight the detrimental effects of free radicals like Reactive Oxigen Species (ROS), are regarded as a major explanation for the preventive role of these diets against most multifactorial diseases [2]. ROS are physiological products of cellular metabolism, and endogenous antioxidant enzymes (such as SOD, catalase and GSH) control their levels leading to a redox homeostasis required for a correct cellular functioning. The redox balance can be altered by uncontrolled elevation of ROS, which, routed antioxidant system defenses, induces injury to lipids, nucleic acids, and proteins, finally causing pathological conditions like cancer, diabetes, cardiovascular and autoimmune diseases [2]. Thus, dietary antioxidants have gained considerable attention for their potential chemo-preventive functions [3–6]. A strong connection between nutrition and prostate cancer is supported by evidence of the large worldwide variations in the incidence of this disease, and by the increased risk of migrants moving from low-risk to high-risk countries [7,8]. In addition, epidemiological studies indicate that obese people develop more aggressive prostate cancer [9,10]. Oxidative stress is one possible link between nutrition and cancer, since high-fat diets increase oxidative stress enhancing proliferation of prostate cancer cells [11]. Resveratrol (RSV), a dietary polyphenol found in high concentrations in grapes, possesses a fascinating wide spectrum of biological activities, with remarkable clinical potential in cardiovascular and neurodegenerative diseases and cancer [12, 13]. Besides its well- recognized antioxidant, anti-inflammatory, growth inhibiting, and pro-apoptotic activities, RSV also mimics caloric restriction [14]. However, despite large efforts, the mechanisms underlying RSV chemo-preventive effects remain still elusive [14,15], mostly because RSV exerts its action through multiple targets, whose downstream effects are dramatically influenced by experimental system, dose, concentration, and duration of treatment. In line with these considerations, the overall conclusion of “Resveratrol 2012” working group was: “There is not yet sufficient evidence to link a specific direct target to a specific health benefit. To date, published evidence from human trials is not sufficiently strong to justify the recommendation of chronic resveratrol consumption by humans for any given indication. The use of resveratrol is not an alternative to maintaining a healthy lifestyle.” [15].

Considering that, for chemoprevention, RSV should be chronically administered to healthy people, the issue of the long term effects of this molecule on normal tissues is crucial. Although the majority of mechanistic studies demonstrate that RSV affects aberrant molecular pathways in tumor cells, in most reports the normal cell counterpart is missing. Moreover, several evidence indicate that also normal cells such as endothelial cells, lymphocytes, smooth muscle cells, chondrocytes, adipocytes, neurons, osteoblasts, hepatic cells, and epidermal keratinocytes [16–19] are vulnerable to RSV. To contribute to this important issue, our interest has been and is to investigate the effects of RSV in non-transformed human cells.

Similar to RSV, also p66Shc, a member of the Shc family of adaptor proteins, is attracting interest [20–22]. Under oxidative stress condition p66Shc undergoes transient phosphorylation at tyrosine residues, and also at Ser36 [22], acquiring redox enzymatic activity that generates ROS in mitochondria. P-Ser36-p66Shc-induced oxidation of cytochrome c contributes to inactivate members of the Forkhead transcription factor family, and down-regulate the expression of antioxidant genes consequently leading to growth arrest and apoptosis [21]. On the other hand, the other two members proteins encoded by the *SHC1* gene, p46Shc and p52Shc, undergo tyrosine phosphorylation in response to cytokines and growth factors, activating the RAS-MAPK pathway and promoting cell proliferation and differentiation. Thus, the three Shc proteins display distinct physiological roles and P-Ser36-p66Shc functions as a dominant-negative regulator of p46/52 Shc by terminating RAS/ERK activation [21]. Despite the fact that many studies imply p66Shc as a mediator of apoptosis, recent studies also associate p66Shc with human epithelial cell proliferation and carcinogenesis. In addition, recent evidence indicates that p66Shc can exert pleiotropic effects on a range of apparently unrelated fundamental biological processes, like cellular adhesion, cytoskeletal morphology and intracellular calcium homeostasis [23–26]. These apparently contradictory results suggest that the biological outcome of p66Shc signaling is more nuanced and might be diverse in different cellular context. Similarly, the canonical, established and distinct roles of the three Shc isoforms are challenged by new results, suggesting that also p52Shc and p46Shc, not only p66Shc, are involved in energy metabolism, and in the metabolic response to over-nutrition and caloric restriction. Interestingly, it has been reported that Shc proteins expression correlates with proliferation of human prostate cancer cells and it is upregulated by steroid hormones in hormone-sensitive cancer cells and in primary prostate carcinomas [27–30].

Strikingly to us, RSV and p66Shc share similar molecular targets involved in the regulation of the same major cellular events, like proliferation and differentiation (FoxO, MnSOD, p27Kip, NF-kB, AKT, ERK, p53, p21) [31–34]. Consistently with these observations, we have previously reported that RSV induces ERK-independent Ser36 phosphorylation of p66Shc in HaCaT cells, a well-known model of human non-transformed keratinocytes [19]. Since RSV and Shc proteins are implicated by differing means in prostate cancer, to get a deeper insight in the effects of RSV on normal epithelial cells and in its association with p66Shc, here we utilized the non-transformed prostate epithelial EPN cell line [35]. Our results show that in EPN cells RSV reduces adhesion and migration, induces growth arrest, stimulates Ser36-p66Shc phosphorylation and abolishes ERK phosphorylation. Finally, our data indicate that RSV profoundly affects non-transformed prostate cells and that its effects may be, at least in part, exerted via a connection with Shc proteins.

## II. METHODOLOGY

### Cell culture and proliferation

EPN cells, spontaneously immortalized prostate epithelial cells, derived from human normal prostate tissue, were obtained in our laboratory [35]. EPN-PKM3 cells have been obtained by transfection of EPN cell with a plasmid bearing PKM, a kinase-negative mutant of PYK2, as previously described [36]. EPN and EPN-PKM3 cells are routinely cultured in Dulbecco Modified Eagle Medium/HAM F12 (DMEM/F12) supplemented with 3% FBS and 1% antibiotics at 37°C, 5% CO_2_, in a humidified incubator. HeLa cells were cultured in DMEM supplemented with 10% FBS and 1% antibiotics at 37°C, 5% CO_2_, in a humidified incubator [37].

### Cell adhesion assay

Adhesion of EPN and EPN-PKM3 cells in the presence or absence of RSV was analyzed by Crystal Violet assay according to Humphries [38]. EPN and EPN-PKM3 cells were seeded at a concentration of 10^5^ in 24 well culture plates and let to adhere for 1, 2 or 4 hours in complete medium supplemented with different concentrations of RSV. For growth assay EPN and EPN-PKM3 cells were seeded in at a concentration of 10^5^ in 24 well culture plates and let to adhere for 18 hours in complete medium, then RSV (25 and 100 µM) was added and cells were cultured for additional 48 hours. At the end of the incubation time cells were washed three times with cold PBS then Crystal violet solution (0,25% crystal violet in 25% methanol) was added for 15′ at room temperature. After de-staining by extensive washing with distilled water, cells were solubilized in 500 μl of 0,1% SDS and OD at 590 nm was determined by spectrophotometry. Adhesion assay were performed in quintuplicate.

### MTT assay

EPN and EPN-PKM3 cells were seeded in 96 well plates in standard medium and let adhere for 24 hours, then standard medium was replaced with standard medium supplemented with different concentrations of RSV (1, 10, 100 and 200 µM) as detailed in the figure legend. After 24 hours CellTiter 96® AQ_ueous_ One Solution Cell Proliferation Assay was performed according to manufactures. Formazan formation was assessed by absorbance at 490nm with a 96-well plate reader.

### Motility assay

EPN cells maintained in standard medium for 48 h were dispersed with versene, washed twice, re-suspended in DMEM/F12 and counted using a hemocitometer. The 24-well modified Boyden chambers, containing porous (8 mm) polycarbonate membranes, were coated, on the internal surface, with 2 mg/cm^2^ P-Lys by incubation at room temperature. The lower chambers were loaded with 500 μl of DMEM/F12 supplemented with 3% FCS, while synchronized cells (2×10^4^) suspended in 200 μl of KSFM 3% FCS, were plated into upper chambers in presence or absence of different treatments as indicated. After 6 h of incubation in 5% CO2 at 37°C, the cells in the upper chamber were removed by a cotton swab, so that only cells that had migrated through the membrane remained. The membranes were then fixed and stained in Coomassie blue solution (0.25 Coomassie blue, 45 ml water, 45 ml methanol, 10 ml glacial acetic acid) for 5 min, then each well was rinsed three times with distilled water. The migrated cells were counted using an inverted microscope.

### Wound healing

EPN and EPN-PKM3 cells, grown to 90% confluence in standard condition, were maintained in serum-free medium for 48 h. The monolayers were scratched with a sterile, disposable 200-ml plastic pipette tip, rinsed twice with phosphate-buffered saline (PBS) and returned to standard medium in presence or absence of 3μM PP2, 20 μMUO126 and 100μM RSV. Then monolayers were photographed after 24 h.

### Western blotting

Cells were grown to sub-confluence in standard medium and serum-starved for 24 hours. Then, medium was supplemented with RSV at varying concentrations or times as indicated in the figure legends. Cells were harvested in lysis buffer (50 mM HEPES, 150 mM NaCl, 1 mM EDTA, 1 mM EGTA, 10 % glycerol, 1 % Triton-X-100, 1 mM phenylmethylsulfonyl fluoride, protease inhibitor cocktail tablet, 0.5 mM sodium orthovanadate, 20 mM sodium pyrophosphate). The lysates were incubated for 30 min on ice and then clarified by centrifugation at 14,000 rpm × 10 min. Total protein concentration was estimated by a modified Bradford assay. For Western blot, 25 or 50 μg/lane of total proteins were separated on 10% SDS-polyacrylamide gel electrophoresis and transferred to PVDF membranes; complete transfer was assessed using pre-stained protein standards. Membranes were blocked in TBS/T buffer (25 mM Tris, pH 7.4, 200 mM NaCl, 0.5% Triton X-100), and 5% non-fat powdered milk for 1 hour at room temperature. Incubation with the primary antibody anti ERK1/2 1:2000, anti-phospho-ERK1/2 1:2000, anti AKT 1:1000, anti phospho-AKT 1:1000, anti p(tyr 317) Shc 1:1000, anti GADPH 1:1000, was carried out overnight in the cold room. Finally, membranes were incubated with the horseradish peroxidase-conjugated secondary antibody (1:3000) for 45 minutes at room temperature and the reactions detected with ECL system.

### IPP procedure

One mg of whole cell lysates from EPN and EPN-PKM3 cells in the various experimental conditions, was immune-precipitated by incubation over night, at 4 °C, with protein A-sepharose-bound anti-Shc polyclonal antibody (4 µl of anti-Shc antibody previously conjugated with 5 mg of protein A-sepharose). Following washings, and resuspension in 30 µl of a standard denaturing protein loading buffer, samples were heated at 70°C for 30 minutes, cleared by a short centrifugation, subjected to 10% SDS-polyacrylamide gel electrophoresis, and transferred to nitrocellulose. Membranes underwent Western Blotting analysis with an anti-phospho-Ser36p66Shc monoclonal antibody (1:1000 dilution; or with an anti-Shc monoclonal antibody (1:1000 dilution) to control for the total amount of Shc isoforms. Finally, the membranes were incubated with the horseradish peroxidase-conjugated secondary antibody (1:3000) for 45 minutes at room temperature and the reactions detected with ECL system.

### Statistical analysis

Data are presented as mean ± SD. Statistical analysis was performed by analysis of variance followed by the Bonferroni test. *P*-values <0.05 were considered statistically significant.

## III. RESULTS

### RSV inhibits adhesion, proliferation and migration of EPN and EPN-PKM3 cells

**Fig. 1.**
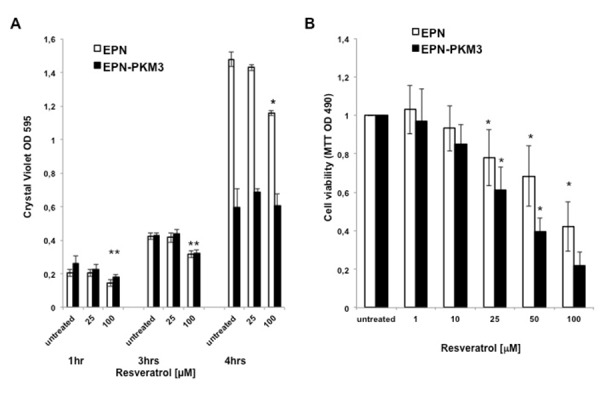
**Effect of RSV on EPN and EPN-PKM3 cells adhesion and viability.** A) Cell adhesion assay. Bars represent the mean of three independent experiments ± SD. Quintuplicate measurements were performed per each experimental point. Statistical analysis was performed by analysis of variance followed by the Bonferroni test (*P < 0.05). B and C) Viability and proliferation of EPN and EPN-PKM3 cells in the presence of increasing concentrations of RSV (MMT assay Fig 1 B, Cristal Violet assay Fig. 1 C). Triplicate measurements were performed per each experimental point. Data represent the mean of three independent experiments ± SD. Statistical analysis was performed by analysis of variance followed by the Bonferroni test (*P < 0.05).

Non-transformed human epithelial prostate EPN cells have been selected as model in which to study the effects of RSV. As a control, we used their counterpart EPN-PKM3 cells, which express a Proline-rich tyrosine kinase (PYK2) dead kinase mutant, and display some tumour-like features, like increased cell motility and migration, and cytoskeleton reorganization [39–42]. PYK2, a non-receptor kinase of the focal adhesion kinase (FAK) family, regulates several cellular functions, like proliferation, apoptosis, actin cytoskeleton organization and adhesion, and its expression inversely correlates with degree of malignancy of prostate cancer [43]. Since cell adhesion is a key event for epithelial cells to survive and proliferate, we investigated adhesion of EPN and EPN-PKM3 cells in the presence or in the absence of RSV. We performed an adhesion assay as described by Humphreis (38), and the effect of RSV on the adhesion of EPN and EPN-PKM3 cells was evaluated at 1, 2 and 4 hours after seeding. RSV (25, 100 µM) reduced EPN and EPN-PKM3 cells adhesion in a dose-dependent manner (Fig. 1A).

**Fig. 2.**
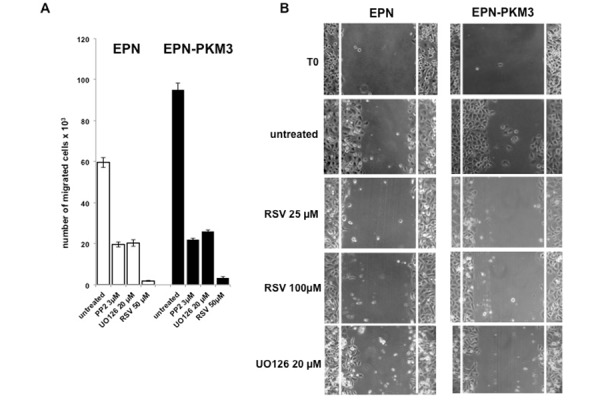
**RSV inhibits migration of epithelial prostate cells**. A) EPN and EPN-PKM3 cells were serum starved for 48 h. Synchronized cells were then plated on polylisine-coated Boyden chambers (as indicated in Methodology) in the absence or presence 3μM PP2, UO126 and RSV. After 6 hours, cells that migrated through and adhered to the under surface of the membranes were fixed, stained, and counted using an inverted microscope. B) EPN and EPN-PKM3 cells monolayers were scrape-wounded and cultured in presence or absence of different compounds, in the absence of serum. The morphology of wound closure was photographed after 24 h.

The effect of RSV on EPN and EPN-PKM3 cells viability and proliferation have been tested by MTT assay and Crystal Violet assay. EPN and EPN-PKM3 cells were seeded in standard medium, and 24 hours later increasing concentrations of RSV (1, 10, 25, 50 and 100 µM) were added for additional 24 hours. RSV reduced viable cells in a dose-dependent manner, and RSV growth inhibitory effect was more potent in EPN-PKM3 cells than in EPN cells (Fig. 1B). Similar results were obtained when cell viability and proliferation were tested by means of Crystal Violet assay (Fig. 1C). Finally, we investigated the effects of RSV treatment on migration, using a modified Boyden Chamber assay, in the absence of extracellular matrix (ECM) proteins (Fig. 2A). EPN and EPN-PKM3 cells were serum starved for 48 h, removed with versene (a non-proteolytic chelating agent), and seeded on poly-lysine coated Boyden chamber. After 6 hours, migration was observed in both EPN and EPN-PKM3 cells and, as expected, it was higher in EPN-PKM3 than in EPN cells. 50 µM RSV treatment was able to drastically abrogate the migration in both cell types, although the magnitude of change was greater in EPN-PKM3 cells. Migration is regulated, among others by c-SRC and MAPK pathways, thus we tested the effect of PP2 and UO126, well-known inhibitor of c-SRC and MEK respectively, on EPN and EPN-PKM3 migration. Interestingly, the anti-migratory effect of RSV was by far more potent than that of PP2 and UO126. Moreover, we assessed migration by wound healing (Fig. 2B). After inflicting a wound, EPN and EPN-PKM3 cells monolayers were extensively washed with PBS and then maintained in serum free medium in the presence or the absence of 25 and 100 µM RSV or 20 µM UO126. After 24 hours, RSV was able to drastically inhibit wound closure at both 25 and 100 µM concentration. The MEK inhibitor-UO126 was less effective than RSV in preventing wound closures in both EPN and EPN-PKM3 cells.

### RSV fails to induce ERK phosphorylation in EPN and EPN-PKM3 cells

**Fig. 3.**
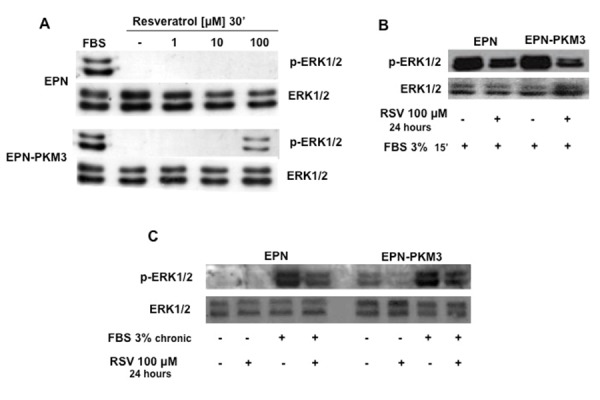
**Effect of RSV on ERK1/2 activation.** A) A representative WB analyzing the dose–response effect of 1, 10 and 100 μM RSV on ERK1/2 phosphorylation in EPN and EPN-PKM3 cells. B) A representative WB analyzing the effect of 24 hours 100 μM RSV pre-treatment on ERK1/2 activation induced in EPN and EPN-PKM3 cells by 15′ minutes stimulation with 3% FBS. C) A representative WB analyzing the effect of 100 μM RSV on ERK1/2 phosphorylation in EPN and EPN-PKM3 cells starved or chronically stimulated with 3% FBS.

The growth inhibitory effect of RSV has been associated to both ERK 1/2 activation and inactivation [44]. To avoid confounding interference of serum on RSV effect, EPN and EPN-PKM3 cells were serum starved for 24 hours before a 30’ treatment with increasing concentration of RSV (1, 10 and 100 µM). We chose a range of RSV concentrations encompassing both doses achievable with diet (1–10 µM) and a pharmacological one (100µM). RSV acute treatment did not induce ERK 1/2 phosphorylation at any concentration tested in EPN cells, whereas a slight ERK activation was observed at 100 µM RSV in EPN-PKM3 cells (Fig. 3A). We then tested whether RSV would interfere with serum-induced ERK 1/2 activation. To this aim, EPN and EPN-PKM3 cells were pre-treated with 100 µM RSV for 24 hours, and then stimulated with FCS for 15 min. As shown in Fig. 3B, RSV was able to strongly reduce FBS-induced ERK 1/2 rapid phosphorylation. Similarly, RSV added to serum chronically stimulated cells also inhibited ERK activation (Fig. 3C). Moreover, we found that RSV did not affect the total amount of ERK 1/2 proteins in all the experimental conditions.

### RSV induces p52Shc tyrosine phosphorylation and p66Shc serine 36 phosphorylation

Shc adaptor proteins are phosphorylated at tyrosine residues Tyr239, Tyr240 and Tyr317 in response to various growth factors and cytokines. The phosphorylated p46 and p52 isoforms transmit signals from receptor tyrosine kinases to the RAS-MAPK pathway, finally inducing mitogenesis. Beside their well-known effect on the regulation of cell proliferation, Shc family members are also involved in the regulation of cytoskeleton rearrangement and migration.

**Fig. 4.**
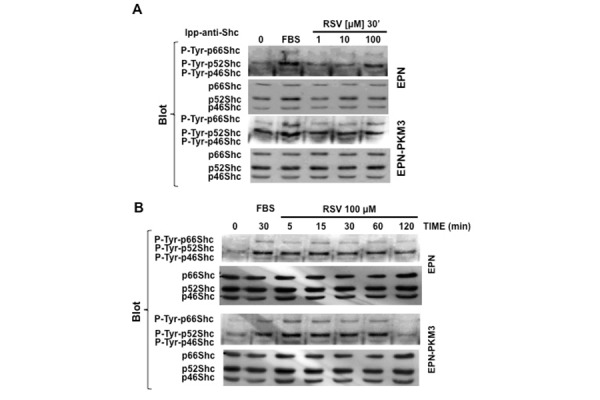
**Effect of RSV on pShc Tyr-phosphorylation.** A) A representative WB analyzing the dose–response effect of RSV (1, 10 and 100 μM) on pShc Tyr317 phosphorylation in EPN and EPN-PKM3 cells. Cell lysates were immunoprecipitated with anti-Shc antibodies prior WB analysis as described in details in Material and Methods. B) A representative WB analyzing the time course of the effect of 100 μM RSV on pShc Tyr 317 phosphorylation in EPN- and EPN-PKM3 cells at 5′, 15′, 30, 60′ and 120′. 3%FCS was used as an internal control. Cell lysates were immunoprecipitated with anti-Shc antibodies prior WB analysis.

On the base of these information, we first tested the effect of RSV on Shc Tyr317 phosphorylation. Acute treatment of EPN and EPN-PKM3 with RSV (1 to 100 µM) resulted in tyrosine phosphorylation of p52 and p66Shc isoforms at 100 µM. These phosphorylations peaked at 15 minutes slightly decreasing thereafter (Fig. 4). Moreover, we did not observe any significant change in the total amount of Shc proteins when the filters were re-probed with an antibody recognizing total Shc proteins.

**Fig. 5.**
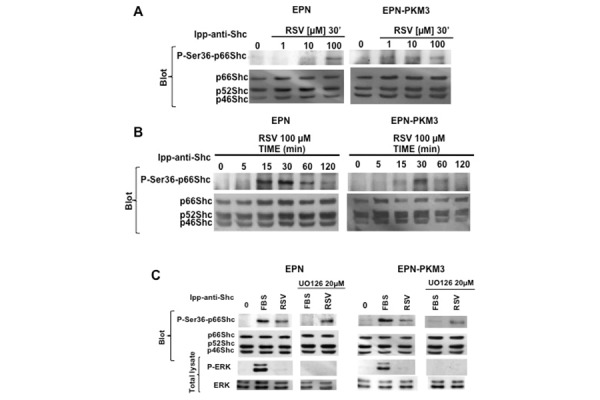
**Effect of RSV on p66Shc Ser36 phosphorylation.** A) A representative WB analyzing the dose–response effect of RSV (1,10 and 100 μM) on p66Shc Ser36 phosphorylation in EPN and EPN-PKM3 cells. Cell lysates were immunoprecipitated with anti-Shc antibodies prior WB analysis.) A representative WB analyzing the time course of the effect of 100 RSV on p66Shc Ser36 phosphorylation in EPN- and EPN-PKM3 cells at 5′, 15′, 30, 60′ and 120′. 3%FCS was used as an internal control. Cell lysates were immunoprecipitated with anti-Shc antibodies prior WB analysis as described in details in Material and Methods. C) A representative WB analyzing the effect of 20 μm UO126 on RSV-induced phosphorylation of ERK and Ser36 p66Shc. EPN and EPN-PKM3 cells were pretreated with 20 μm UO126 for 6 h followed by 30′ treatment with 100 μm RSV. As a control, cells were stimulated with 3% FCS after U0126 pretreatment. Cell lysates were immunoprecipitated with anti-Shc antibodies prior WB analysis as described in details in Material and Methods.

p66Shc appears to be functionally different from the p46 and p52 isoforms, and, although it is tyrosine-phosphorylated, Ser36 phosphorylation seems to be the main regulator of its activation. When EPN and EPN-PKM3 cells were acutely stimulated with increasing concentration of RSV (1 to 100 µM), a dose-dependent increase of the levels of P-Ser36-p66Shc was observed (Fig. 5A). However, EPN and EPN-PKM3 display a different sensitivity to the stimulating activity of RSV. In fact, P-Ser36-p66Shc phosphorylation is induced at diet achievable concentrations (1 and 10 µM) in EPN-PKM3 cells, and at pharmacological doses (100 µM) in EPN cells. P-Ser36-p66Shc phosphorylation peaks at 30 minutes upon 100 µM RSV stimulation and declined at 120 min in both EPN and EPN-PKM3 cells (Fig. 5B).

ERK has been implicated in p66Shc phosphorylation in response to endothelin, taxol and H_2_O_2_ [45, 47]. In addition, Suen and co-workers reported a direct interaction between p66Shc and ERK [48]. To further investigate whether in EPN and EPN-PKM3 cells RSV effect on p66Shc is independent on ERK, we tested the effect of MEK inhibitor UO126 on serum- and RSV-induced p66Shc Ser36 phosphorylation. UO126 wipes out serum-induced P-Ser36-p66Shc, but it fails to affect RSV-induced Ser-36 phosphorylation (Fig 5C). These data suggest that, in our experimental setting, RSV induces Shc tyrosine phosphorylation and p66Shc Ser36 phosphorylation in an ERK-independent manner.

### RSV is not able to induce Ser36-p66Shc phosphorylation in HeLa cells

**Fig. 6.**
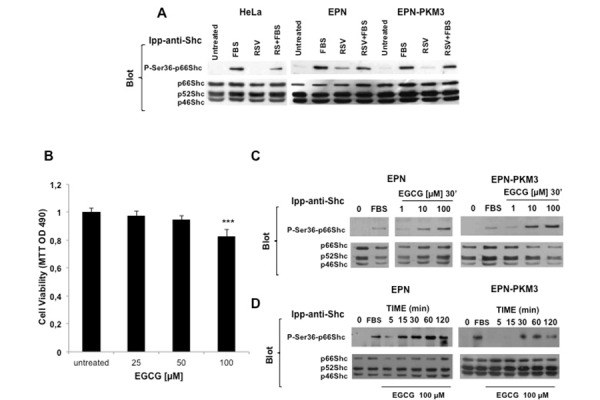
**Effect of RSV on p66Shc Ser36 phosphorylation in HeLa Cells, and effect of EGCG on EPN cells viability and on p66Shc Ser36 phosphorylation in EPN and EPN-PKM3 cells.** A) A representative WB analyzing the effect of acute treatment of 100 μM RSV on FBS-stimulated Ser36-p66shc phosphporylation in serum starved HeLa, EPN and EPN-PKM3 cells. Cell lysates were immunoprecipitated with anti-Shc antibodies prior WB analysis. B) The effect of EGCG (25, 50 and 100μM) on EPN cell viability. Triplicate measurements were performed per each experimental point. Data represent the mean of three independent experiments ± SD. Statistical analysis was performed by analysis of variance followed by the Bonferroni test.*P < 0.05. C) A representative WB analyzing the dose–response effect of EGCG (1,10 and 100 μM) on p66Shc Ser36 phosphorylation in EPN and EPN-PKM3 cells. Cell lysates were immunoprecipitated with anti-Shc antibodies prior WB. D) A representative WB analyzing the time course of the effect of 100 EGCG on p66Shc Ser36 phosphorylation in EPN- and EPN-PKM3 cells at 5′, 15′, 30, 60′ and 120′. 3%FCS was used as an internal control. Cell lysates were immunoprecipitated with anti-Shc antibodies prior WB.

To test whether the ability of high concentration of RSV to induce Ser36-p66Shc phosphorylation is a common effect of the phytoalexin on any cell type, we tested the relation between RSV and p66Shc also in HeLa cells, a well-recognized model of cervical cancer in culture [37]. Over-night serum starved HeLa cells were pretreated for 30 min with FBS, and then stimulated for 15 min with RSV 100 µM. Serum induced Ser36-p66Shc phosphorylation (Fig. 6A), whereas RSV had no *per se* effect on Ser36-p66Shc phosphorylation in HeLa cells. However, RSV decreased serum-induced p66Shc Ser36 phosphorylation.

### EGCG regulates EPN cell proliferation and induces p66Shc serine 36 phosphorylation

Finally, to investigate whether the ability of RSV to induce Ser36p66Shc phosphorylation is a common feature of other antioxidant molecules, we tested Epi-gallo-catechin Gallate (EGCG), the major and the most active component of green tea and of the Asian diet. EGCG exerts anti-oxidant and anti-inflammatory effects protecting from DNA damage, LDL oxidation, lipid peroxidation, oxidative stress and production of nitric oxide (NO) radicals, the latter effect due to the inhibition of the expression of iNOS. EGCG also lowers the overproduction of pro-inflammatory cytokines and mediators, reduces the activity of NF-kB and AP-1, and the subsequent formation of peroxynitrite, NO and ROS. Green tea is proposed to be a dietary supplement for the prevention of cardiovascular diseases, in which oxidative stress and inflammation are the principal causes after the establishment of hyperlipidemia. Additionally, the cancer-preventive effects of green tea are widely supported by results from epidemiological, cell culture, animal and clinical studies [49–51].

*In vitro* cell culture studies show that tea polyphenols potently induce apoptotic cell death and cell cycle arrest in tumor cells but not in their normal counterparts [52]. Here, we found that treatment of EPN cells with increasing concentration of EGCG (25, 50 and 100 µM) results in a dose-dependent inhibition of cell proliferation. The inhibitory effect of EGCG was observed after 48 hours and was more pronounced at the higher concentration (100 µM) (Fig. 6B). Of note, the inhibitory effect of EGCG was by far lower than that of RSV at the same concentrations. On the other hand, at the same concentration tested for RSV (1 to 100 µM), EGCG was able to induce phosphorylation of p66Shc in Ser36 (Fig. 6C and 6D).

## IV. DISCUSSION

The effect of RSV on normal cells is a key issue for a safe exploitation of the clinical potential of this molecule in humans. In the present work, we report that RSV influences different functions in EPN cells, a model of non-transformed prostate epithelial cells, and in their counterpart bearing a kinase-negative mutant of the non-receptor kinase PYK2. In particular, RSV inhibits cell adhesion, cell migration and cell viability of EPN and EPN-PKM3 cells. Well aware of conclusions and recommendation of “Resveratrol 2012” working groups, we have used concentration of RSV both in the range achievable with diet (1nM to 25 µM) and in the pharmacological one (50 to 100 µM). In EPN and EPN-PKM3 cells adhesion is significantly reduced at 100 µM RSV, whereas both viability and migration are already reduced at 25 µM. In addition, migration assays indicate that the anti-migratory effect of RSV is more potent than that of PP2 (SRC inhibitor) and UO126 (MEK inhibitor). To trace back the molecular bases of inhibitory action of RSV, we have analyzed its effect on the members of the Shc family adaptor proteins. In EPN and EPN-PKM3 cells, RSV induces a time- and dose-dependent phosphorylation of Shc proteins. In particular, RSV increases p52Shc and p66Shc tyrosine and p66Shc Ser36 phosphorylation.

Moreover, RSV fails to induce ERK phosphorylation, and decreases serum-induced ERK activation, in both acute and long-term treatment. Since a physical and functional interaction between ERK and Shc proteins has been described, we tested the role of the MEK inhibitor UO126 on p66Shc phosphorylation. UO126 wipes out serum-induced, but not RSV-induced, Ser36-p66Shc phosphorylation. Interestingly, the effect of RSV on p66Shc activation is apparently dependent on the cellular context, since in HeLa cells RSV fails to induce p66Shc Ser36 phosphorylation. Conversely, activation of p66Shc phosphorylation is not restricted to the sole RSV, since it is also induced by EGCG, another well-known food antioxidant, in EPN and EPN-PKM3 cells.

The data presented here suggest that, in our experimental setting, Shc phosphorylation does not correlate with cell proliferation, neither in the case of RSV nor in that of EGCG. The estrogen-like behavior of RSV is one possible explanation of this observation. Intriguingly, there are multiple connections between estrogens, RSV and Shc proteins: RSV binds estrogens receptors; α5ß3 integrin, recognized as RSV receptor, presents specific binding sites for steroids [53,54]; estrogens induce p52Shc activation [55,56] which, in turn, has been identified as a primary signaling partner for the tyrosine(s)-phosphorylated cytoplasmic tails of activated α5ß3 integrin [57]. However, other molecular pathways connecting RSV and SHC such as calcium regulation can not be ruled out.

Beside its proapoptotic role, p66Shc activation is associate also to the regulation of cell proliferation, migration, cytoskeleton rearrangement, differentiation, senescence and, last but not least, autophagy. Thus, these non canonical p66Shc effects may represent one of the possible mechanisms through which RSV controls prostate epithelial cells functions. Within the context of our experimental setting, it is possible to speculate that RSV-induced p52Shc tyrosine phosphorylation drives p66Shc Ser36 phosphorylation which, competing with p46Shc and p52Shc for Grb2 binding, inhibits p52Shc mediated RAS-MAPK proliferative stimulus and consequently induces growth arrest, inhibits cell migration and motility. Noteworthy, although RSV induces “qualitative” same effecs in EPN and EPN-PKM3 cells, from the “quantitative” stand point the effects differ in the two cell line. In that, RSV achieves the same effect in EPN-PKM3 cells at lower doses than in EPN cells, on the other hand at the same dose the extent of RSV effects differs in the two cell lines.

In conclusion, the data presented here indicate once more that in a model of non-transformed epithelial cells, RSV induces growth arrest, modification of enzyme activity and/or cell signaling pathways. For this reason, before RSV could be safely recommended for its potential beneficial effects on human health, its effect on normal cells need to be extensively investigated.
